# Key issues in the effectiveness of public financial tools to support childbearing the example of Hungary during the COVID-19 crisis

**DOI:** 10.1371/journal.pone.0273090

**Published:** 2022-08-16

**Authors:** Judit Sági, Csaba Lentner

**Affiliations:** 1 Department of Finance, Faculty of Finance and Accountancy, Budapest Business School, Budapest, Hungary; 2 Széll Kálmán Public Finance Lab, Faculty of Governmental and International Studies, University of Public Service, Budapest, Hungary; University of Pecs Faculty of Humanities: Pecsi Tudomanyegyetem Bolcseszettudomanyi Kar, HUNGARY

## Abstract

The propensity to have children, which, according to the view accepted in the literature, is a good predictor of actual childbearing, is of particular importance in countries with low fertility rates and economic prosperity. In this paper, we report the results of a representative survey of 15,700 respondents in 2021 of university students in an emerging market economy in Central Europe, mapping their intentions to have children. The PLS-SEM data analysis method was used to test our hypotheses on the relationships between social, economic, and environmental variables of childbearing. Our results confirm the dominant role of socio-cultural inclusiveness in childbearing, over socio-economic and environmental-economic factors. The novelty of our research lies in the impact analysis of family policy incentives; however, our results are consistent with those documented in the literature, namely, the primacy of socio-cultural factors in the willingness of childbearing.

## Introduction

In our study, we look at a European country (Hungary) where the population has been declining for many decades (steadily over the last four decades). Meanwhile, after the change of regime, the living standards of the population have improved markedly and macroeconomic indicators have stabilized; however, there has been no improvement in population statistics: birth rates have remained low and emigration has persisted. The rate of natural decrease in the population has remained stable at an annual rate of over 4% in recent years (according to The Hungarian Central Statistical office-KSH) [1]. This has been due to low fertility rates and high mortality rates, particularly during the pandemic. For these reasons, economic policy has increasingly focused on increasing the number of marriages and births.

In many European countries, including Hungary, policymakers use fiscal policy instruments to encourage childbearing. This includes direct income transfers to parents raising children, usually increasing with the number of children, tax credits or tax allowances, or housing subsidies. The novelty of our research lies in the assessment of the impact of family policy incentives: to the authors’ knowledge, no survey has yet examined the propensity to have children, including both tax and housing elements of family policy incentives.

In 2021, the following pro-birth policy elements were available in Hungary for families with children:

- family tax and social contribution allowance, which reduces the overall tax base of taxpayers,
- From 1 January 2020, personal income tax exemption for mothers with four or more children,
- Infant care allowance for maternity leave, and thereafter childcare allowance and family allowance,
- the Housing Allowance for Families (Henceforth: CSOK) introduced in 2015 and extended in 2016, and the 3% interest subsidised CSOK loan that can be taken out for it,
- the mortgage loan waiver and student loan waiver schemes launched in 2018 and extended in 2019,
- the 0% baby loan launched on 1 July 2019, the rural CSOK and the subsidy for large families to buy a new car,
- the home renovation subsidy starting on 1 January 2021 and the 3% subsidised home renovation loan for advance payment starting on 1 February, as well as
- a housing tax rebate and exemption from fees for CSOK applicants, which will also be available from 2021, and a CSOK for loft conversions and floor extensions,
- the Green Home Programme with a fixed interest rate of 2.5%, available from 4 October 2021, and the interest-free green CSOK loan available under this programme.

In practice, a family with three or more children is not required to pay personal income tax and pension contributions through regular income transfers (assuming an average income level). Through the one-off (non-recurring) grants, most of which facilitate access to housing, provide non-repayable grants of up to almost half the price of a new dwelling, above which a subsidised loan can be claimed, however, we see significant differences between the capital and large rural cities, as well as less frequented, deprived rural areas. For the year 2021, all parents entitled to the family allowance have received a refund of the personal income tax paid.

Non-regular forms of family support also differ according to which children they are available for, such as the CSOK, the village CSOK and the related interest subsidised loans for both existing and unborn children, while mortgage loan relief and student loan relief are only available for children. The baby loan is available to those who will have at least one child within 5 years. The renovation grant, the related interest subsidy loan and the large family car purchase grant are only available for existing children.

In our study, without going into the details of the forms of support granted to families with children, we intended to raise attention to the diversity of support and the importance of the policy maker’s intention to encourage childbearing. The focus of our study is on how family policy incentives affect the propensity to have children. Childbearing decisions are imperfectly predicted by childbearing propensity, but they do predict it; reflecting the combined effects of natural fertility and the circumstantial constraints [1].

The next chapter of our study will review the literature on childbearing in order to derive our hypotheses. The following section presents the aim of the study (i.e. how effective are the state incentives in influencing the childbearing intentions of the Hungarian youth) and the methodology and results of our representative, self-administered questionnaire survey among university students. This is followed by our conclusions and recommendations, placing the issue of pro-birth policy elements and intended childbearing in a broader social context.

## Theoretical perspective

### Socio-cultural inclusiveness of childbearing

In the international literature, research documenting the propensity to have children dates back to the 1950s, with the first models typically examining fertility in its biological context [2]. In addition to the biological determinants of fertility (conception rate, risk of miscarriage, and the age patterns of natural fertility of married women), the early research focused on different modes of birth control [3–5]. A key finding is that, in addition to biological endowments, fertility is ultimately determined by factors at the family level, including the extent to which marriage (the family) provides a positive, supportive environment for childbearing [6, 7].

Bongaarts [8] and Easterlin and Tilly [9] place the natural, intended and actual fertility rates in the classical supply-demand model. Natural fertility is the supply variable in the model: it refers to the maximum number of children that could be born without contraception and abortion. Family planning plays the role of demand for childbearing as a factor that overrides natural fertility, reducing it to the planned number of children. We cannot ignore the fact that the social environment has a major influence on the values of the family, in terms of having and raising children, as the demographic revolutions of the 20^th^ century have shown.

Caldwell [10], examining the demographic transitions in the developed world, concludes that during the first demographic transition, the one-child family model became accepted in society. Social perceptions [11] of having a second child, and the intra-family factors (income, education) [12, 13] associated with having a second child, reduced the intended fertility rate, while the world wars led to further population decline.

During the second demographic transition (typically in the 1960s), cohabitation outside marriage became more common and the proportion of children born out of wedlock continued to rise [14]. A shift in social values was observed: family values were increasingly replaced by individual values [15], income and wealth relations shifted as women entered the labour market [16], the spread of extra-marital cohabitation led to a dissolution of marriage and the traditional family model, the postponement of childbearing [17] and a decline in fertility [18].

The change in union (marriage) dynamics alongside individualization can be traced nowadays as well. In a recent study Billingsley and Oláh [19] examined co-residential partnerships in post-socialist countries, and found that in Central-European countries, especially in Hungary, the number of years spent in a co-residential union before the age of 30 years decreased, which–perceived as partnership instability–may have contributed to decreasing fertility among them.

Based on this, the support for childbearing within the family and the social environment of the family can be considered as the primary factors in the examination of the willingness to have children. *Our first hypothesis (H1) is that the emotional (i*.*e*. *relationship)*, *cultural and health background within the family has the same (positive) influence on the willingness to have children*.

### Socio-economic factors

According to research by Van Roode et al. [20], socioeconomic factors play the strongest role in the timing of having a first child; they found that individuals with higher education and higher income levels delay the timing of having a child.

In the Economic Theory of Fertility Decline, [21, 22], the marginal utility of having children is reduced because of the loss of household income. Carrying a child, giving birth, and usually during the first years of the child’s life, the mother is partially or completely excluded from the labour force; her return to the labour market is also in doubt if she has more children [23]. It is questionable whether the father’s earnings can compensate for this loss of income and what the family situation requires of the father. In the theory of family (household) economics (New Home Economics, [24, 25], the household’s wealth, labour supply and accumulated wealth determine the financial possibilities of having children.

The Theory of the Allocation of Time [26] also highlights the relative costs of having children, in that time spent raising children takes time away from parents’ existing limited time, thereby increasing the value of leisure. The availability of financial resources to spend on children and the satisfaction associated with having children theoretically determines an ideal number of children for a given family, at least in the model of quality childbearing [27–29].

Gietel-Basten and Verropoulou [30] found that even in a society based on a traditional family model (where marriage and childbearing are linked), the opportunity cost of the marriage package has become too high because of the learning opportunities and career paths that women nowadays have to take. Whereas, the rejection of traditional values and the inclination for self-expression can have a stronger correlation with the acceptance of voluntary childlessness as well [31].

Prag et al. [32] point out that women’s socio-economic status (measured by education and professional position) highly influences childlessness, and that in Central and Eastern European countries the low but increasing voluntary childlessness could be related to the socioeconomic (i.e. labour market) and cultural transformations of the previous decades. The post-communist fertility transition, investigated by Spéder and Kapitány [33] has been characterized by worse chances of realizing intentions for giving birth, especially among women after the turn of the millennium.

On the basis of the above, we formulated our *second hypothesis (H2)*, *i*.*e*. *that the financial costs of having children within the family* (changes in income as a result of having children, including the additional burden of child-rearing, the mother’s lost income and other additional costs, as well as sacrifices in career and leisure) have a negative impact on the willingness of childbearing.

### Environmental(spatial)-economic factors

Robinson’s study [34] has shown that the incentive or disincentive effect of the social environment and the family’s financial resources alone only partly explain the propensity to have children; in addition, it is reasonable to take into account, for example, spatial factors in fertility trends. According to Kulu et al. [35], the likelihood of having a first child is lower for people living in large cities. Actual fertility rates tend to be higher in the outer periphery of large cities, especially in rural areas [36], although this is also due to the effect of families with children already living in such areas.

In addition to the number of existing children and the mother’s age, marital status, parents’ education and employment status, housing conditions and the number of previous moves play a role in the polarisation of the propensity to have children [37–40]. Similarly, the availability of day-care facilities for young children and the public day-care nursery system also affects parents’ decisions about the number of their children [41].

According to the Contextual hypothesis and the Selection hypothesis proposed by Basten et al. [42], a child-friendly living environment (suburban house, availability of childcare services and infrastructure and affordable costs) encourages childbearing. Housing choice criteria and housing conditions usually reflect the social status of parents [36, 43, 44].

In view of this, *our third hypothesis (H3) is the following*: *the infrastructure of the residential environment and the employment opportunities associated with the place of residence have the same (positive) effect on the willingness to have children*.

### Pro-birth policy elements

The study by Song et al. [45] points out that, in our time, women of childbearing age, in particular, need to improve their attitudes towards marriage and childbearing in order to see a meaningful change (increase) in the number of children they have. To this end, it proposes the use of family policy incentives and awareness-raising through social media.

In the references (as far as we know), Billingsley and Ferrarini [46] were the first to raise the question of how family policy incentives can influence the propensity to have children. In their 2014 study of European countries, they found that any form of family support has a positive effect on having a first child and on having subsequent children (allowing women to participate in the labour market, according to the earner-carer model).

Reflecting to the characteristics of the Central and Eastern European countries, Spéder et al. [47] pointed at the signalling effect of specific policy measures in the benefit of parents with three or more children, as a recognition of stay-at-home motherhood.

The effect of pro-birth policy elements can be assessed more accurately if we consider the changes in age-specific fertility rates: if these rates were disproportionately lower than expected among women in their later twenties (as it was the case of Hungary), then the demographic regime is described by a bimodal fertility curve, i.e. the co-existence of an early and a late childbearing [48]. Likewise, the incentives for childbearing exercise their effects differently across educational levels as well. The health, social, welfare and economic impacts of the COVID pandemic are being detected in many areas, and are likely to have had and will continue to have an impact on birth rates. Ullah et al. [49] suggest that there is likely to be an initial decline in fertility rates, but that this effect will reverse in the short term. The impact of fiscal policies in crisis management has been felt through the operation of health care systems, income policies and family policy incentives. Using US hospital data, Stout et al. [50] found an initial decline in the number of births associated with the social changes of the COVID-19 pandemic, followed by an expected growth in it after the lockdown.

**Fig 1:**
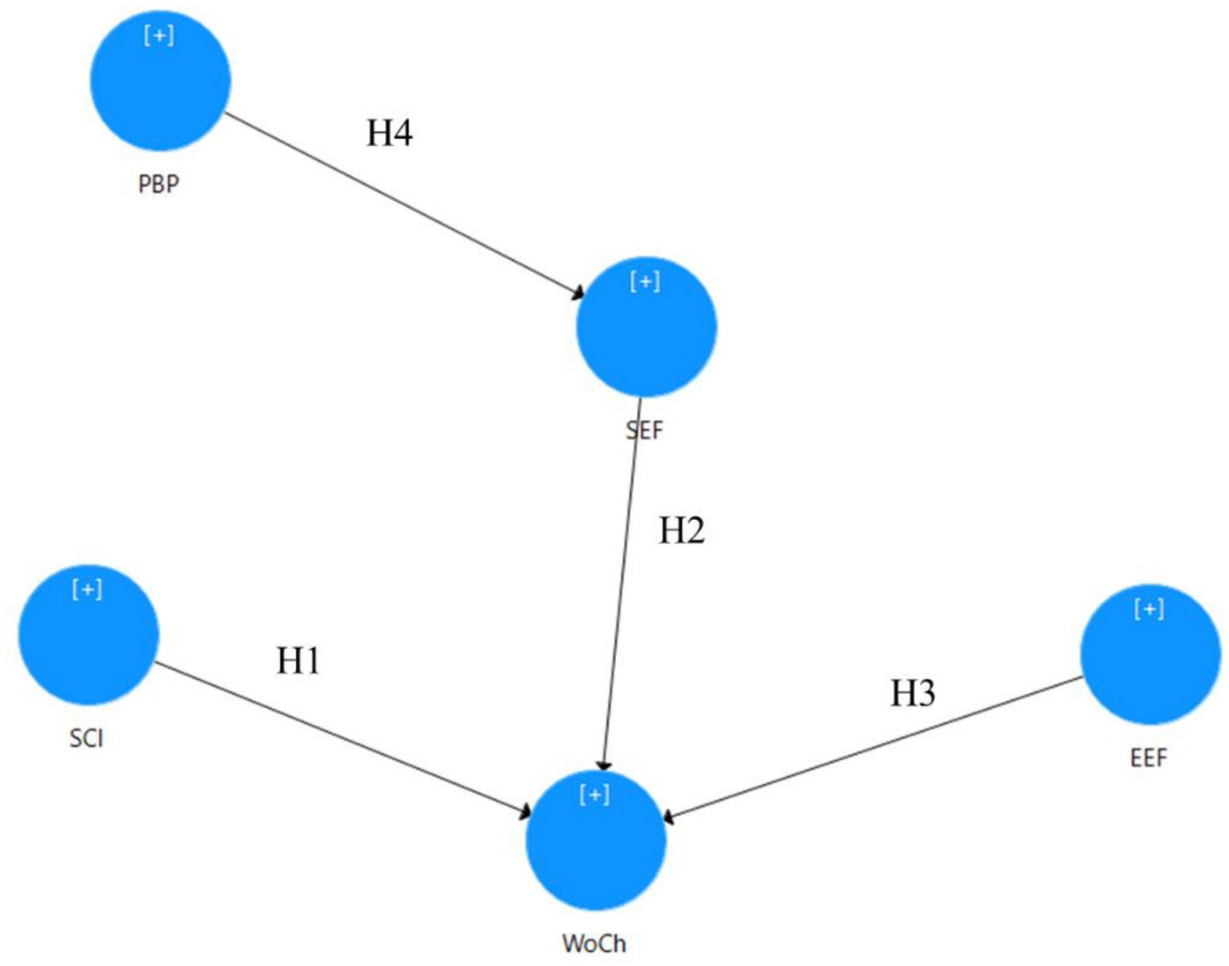
Hypotheses development.

*Our fourth hypothesis (H4) is that state financial instruments and subsidies supporting childbearing have a positive impact on the financial development of childbearing within the family and*, *indirectly*, *on the willingness to have children*. Fig 1 shows the four hypotheses as described before.

SCI refers to socio-cultural inclusiveness of childbearing; SEF refers to socio-economic factors; EEF refers to environmental-economic factors; PBP refers to pro-birth policy elements; and WoCh refers to willingness of childbearing.

## Objectives of the study

Many countries in the developed world are struggling with the decrease of the propensity to have children and fertility rates. Forecasting the birth rate is important for health care systems and government agencies to plan the level of intervention, to model the active and/or ageing population and thus to maintain the performance of the economy in terms of the expected active labour force.

Countries with adequate public resources make extensive use of family-type tax credits and direct financial support, while also seeking to improve the community infrastructure for child-rearing. In the case of Hungary, pro-birth policies include both regular income transfers and non-systemic (mostly housing) subsidies. However, a growing body of international research shows that childbearing is not fundamentally dependent on the financial situation of the family, and thus the impact of state subsidies on childbearing is also assumed to be indirect.

The research seeks to answer the question of how young Hungarian university students thought about having children in the context of the COVID-19 pandemic crisis—in the light of an empirical study. Due to the representativeness of the sample (i.e. Hungarian university students represent more than one fifth of the total population of their age group), the results can be extended with good approximation to Hungarian families as well.

## Data and methodology

The authors declare that the present study has been reviewed and approved by the Vice-Rector of the University of Public Service (Hungary, Budapest) on behalf of the University’s management. This written consent has been issued before the study began and stated that "The Vice-Rector was aware of the research agenda of the study, and—upon guidance of the ethical standards in research processes, and in accordance with the principles expressed in the Declaration of Helsinki—approved the research on the elements and effectiveness of pro-birth policy in Hungary and gives way to the execution of the research under regular (quarterly) monitoring". Upon completion, the Vice-Rector–based on the final version of the study–confirmed that the research has been conducted according to the ethical research standards in all phases of the research process.

The authors carried out their own questionnaire survey on the intention to have children among young people of ’normal’ age to start a family; among university respondents. This questionnaire had a confidentiality and personal data protection clausure, stating that the data on individuals may be used in aggregate and only for the purposes of the given willingness of childbearing research and that only the Authors could have access to these data in order to prevent the disclosure of information on any individual. There were no minors among the responders, as we distributed our questionnaire only among university students (that is, from above the age of 18 years). Responses were collected in January-March 2021 and 15,700 students of higher education at 20 of Hungary’s universities with the largest student population were asked. The statistical characteristics of the respondents are presented in Table 1.

**Table 1:** Statistical characteristics of the respondents.

	N	%
Gender		
Male	6074	39%
Female	9626	61%
Field of education		
Economics	3731	24%
Engineering, IT	3548	23%
Other	8421	54%
Age		
Less than 20	2549	16%
20 to 25	11824	75%
25 to 30	1175	7%
Above 30	152	1%

The 20 universities included in the survey have a total of 239,785 full-time, distance, evening and correspondence students (data for the academic year 2020/21, [51]), representing 84.1% of the 285,110 total university students in Hungary. The survey was also representative of young adults with high expected future incomes who are enrolled in higher education (they represent 21.3% of the total population of this age group).

From the results of our earlier survey of similar university students in 2018, we concluded that a relatively high proportion of young people do not yet want children (they postpone having children). Among the main reasons given are difficulties in finding a suitable partner/spouse and insufficient social services related to children (crèches, nurseries, child protection services, etc.) were cited [52]. Our previous research has also indicated that although fiscal policy supports the purchase and construction of housing associated with having children, the amount of housing subsidies is not high enough to provide a measurable incentive to have children at the national level, given that these young adults will seek work and housing in more developed larger cities where housing is increasingly expensive [53]. The rise in Hungarian house prices has been the second largest in the EU in the last decade (up 118%) [1].

In our present questionnaire research, we examined broader aspects of willingness to have children. Four questions were asked about the motivations for having children in the context of their immediate living environment (environmental-economic factors, EEF); respondents were asked to indicate on a scale of 1 to 10 how reluctant (1) or committed (10) they were to have children, taking into account the infrastructure of their living environment and the employment opportunities available in their place of residence. A further four questions focused on the effectiveness of public financial instruments and subsidies (pro-birth policy elements, PBP) in supporting childbearing, asking respondents to indicate on a scale of 1 to 10 the extent to which they thought the government had improved the living conditions of families who had children. Eight questions were asked about the socio-cultural inclusiveness (SCI) of having a child within the family, asking respondents to indicate on a scale of 1–10 the extent to which their immediate environment supports them in having a child. Finally, five questions were asked to assess respondents’ socio-economic factors (SEF) in relation to their family background for having children, measuring aspects of career and income on a scale of 1–10. Intention to have children was assessed for three time horizons (short, medium and long term), as recommended by Brzozowska and Beaujouan [54].

Partial least squares structural equation modelling (PLS-SEM) data analysis method is used to test our hypotheses on the relationships between variables. A structural model is developed to show the constructs and the path relationships between them, and how the latent variables are related to each other. The method builds on the characteristics of the indicators and relationships in our research model. The use of this method is justified in empirical studies where the aim is to explore complex relationships between dependent and latent variables [55]. The estimation procedure for PLS-SEM is an ordinary least squares regression-based method, which uses available data to estimate the path relationships in the model with the objective of minimizing the error terms (i.e., the residual variance) of the endogenous constructs. In other words, PLS-SEM estimates coefficients (i.e., path model relationships) that maximize the R^2^ values of the (target) endogenous constructs [56]. In writing the present study, Smart PLS 3.3.5 software was used to set up the research model from the hypotheses.

## Results

The analysis and interpretation of PLS results consists of two main stages; the measurement model and the structural model. The first stage determines whether the indicators and constructs have been measured correctly (*outer model*) and the second stage determines whether the relationships between the constructs are significant or not.

In the measurement model, we estimate the reliability and validity of the variables. The results reported in Table 2 show that all variables (constructs) meet the requirements of Cronbach’s alpha, Dijkstra-Henseler rho (rho_A) and composite reliability, as the values are above the critical level of 0.7, indicating that all variables have convergence or internal consistency. Finally, it is observed that all constructs meet the minimum criterion of 0.5 points required by the average variance extracted (AVE), meeting the convergent validity of the constructs and dimensions criterion [57].

**Table 2:** Results of the measurement model analysis.

	Loading	Cronbach’s Alpha	rho_A	Composite Reliability	Average Variance Extracted
**Environmental-economic factors (EEF)**		0.977	0.978	0.983	0.937
EEF_1a	0.157				
EEF_1b	0.153				
EEF_2a	0.146				
EEF_2b	0.153				
**Pro-birth policy elements (PBP)**		0.968	0.972	0.977	0.913
PBP_1a	0.153				
PBP_1b	0.164				
PBP_2a	0.174				
PBP_2b	0.166				
**Socio-cultural inclusiveness (SCI)**		0.945	0.947	0.955	0.725
SCI_1a	0.640				
SCI_1b	0.599				
SCI_2a	0.576				
SCI_2b	0.582				
SCI_3a	0.602				
SCI_3b	0.575				
SCI_4a	0.663				
SCI_4b	0.635				
**Socio-economic factors (SEF)**		0.741	0.979	0.800	0.575
SEF_1a	-0.021				
SEF_1b	-0.016				
SEF_2a	0.186				
SEF_2b	0.177				
SEF_2c	0.189				
**Willingness of childbearing (WoCh)**		0.946	0.950	0.965	0.903
WCL	0.918				
WCM	0.972				
WCS	0.960				

Table 3 presents the results of the discriminant validity test, following the Fornell-Larcker criterion. Since for all variables (constructs) the cross loading (square root of the AVE) is higher than the values within the corresponding construct, it can be concluded that all variables (constructs) are consistent, acceptable and confirmed by the Fornell-Larcker criterion. From the analysis, we conclude that we have a reliable and valid measurement model.

**Table 3:** Discriminant validity: Fornell-Larcker criterion.

	**EEF**	**PBP**	**SCI**	**SEF**	**WoCh**
**EEF**	**0.968**				
**PBP**	-0.007	**0.955**			
**SCI**	-0.009	-0.027	**0.851**		
**SEF**	-0.007	0.487	-0.013	**0.759**	
**WoCh**	0.157	0.172	0.717	0.188	**0.950**

To test the hypotheses, the structural model was bootstrapped with 500 subsamples [57]. The significance and correlation of each hypothesized pathway and the explained variance are important for the analysis of the structural model. The model explains 58% of the variance in the willingness of childbearing. The path coefficients and the correlations between the variables (constructs) are shown in Table 4, as follows:

**Table 4:** Hypotheses testing.

		Original Sample (O)	Sample Mean (M)	Standard Deviation (STDEV)	T Statistics (|O/STDEV|)	P Values	Status
**H3**	**EEF -> WoCh**	0.165	0.165	0.006	29.223	**0.000**	**Accepted**
**H4**	**PBP -> SEF**	0.487	0.488	0.006	75.563	**0.000**	**Accepted**
**H1**	**SCI -> WoCh**	0.721	0.721	0.005	150.276	**0.000**	**Accepted**
**H2**	**SEF -> WoCh**	0.198	0.198	0.006	34.934	**0.000**	**Accepted**

Our results show that both the living environment (environmental-economic factors), public financial instruments (pro-birth policy elements), socio-cultural inclusiveness and financial background (socio-economic factors) have a positive and significant impact on the willingness of childbearing. Consequently, our hypotheses on the propensity of young adults to have children are accepted.

In terms of the strength of the effect on childbearing intentions, we find that only Socio-cultural inclusiveness of childbearing has a strong effect, while Socio-economic factors and Environmental(spatial)-economic factors are not main determinants of childbearing intentions. Although Pro-birth policy incentives have a moderate effect on the financial implications of childbearing, their effect on the willingness of childbearing is small.

After examining the causal relationships in more detail, we conclude that the effects of all variables are positive and significant, except for leisure time and career. Neither the loss of leisure time (SEF_1b) nor the reduction in career opportunities (SEF_1a) was considered by our respondents to be an important factor in their intentions to have children, which can be explained by the support of the wider family (grandparents, and siblings), the additional state support linked to having children, and the emergence of increasingly family-friendly jobs [58]. Further research is needed to examine these links, which has not been the purpose of this study.

**Fig 2:**
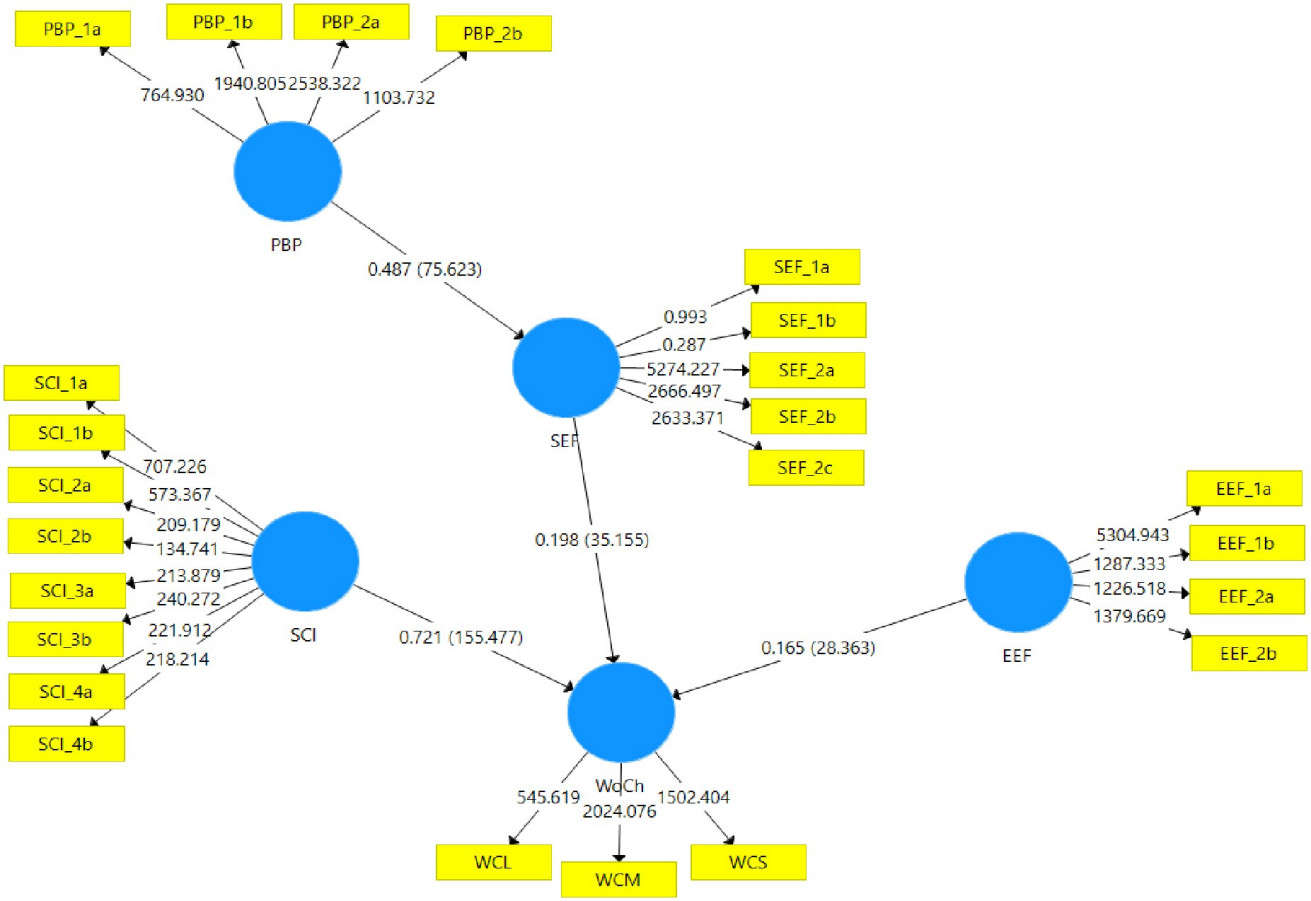
Hypotheses test results.

Fig 2 shows the results of the hypotheses on the relationships between the variables, with R squared values and t-statistics (T-values) indicating the direction and strength of the relationship between the model explanatory variables.

## Discussion

The study by Brzozowska et al. [59] highlights the differences in attitudes towards childbearing in Eastern and Western European countries and how these effect reproductive behaviour. For example, differences are reflected in the fact that short-term childbearing propensity is lower in post-socialist countries, while the proportion of unplanned pregnancies or later planned births is higher. In our view, in post-socialist countries, including Hungary, democratic institutions have not been consolidated to the extent that young adults who are sexually active and capable of having children have a stable vision of the future in which having children is considered to be a feasible option. Unfortunately, the pandemic has amplified these factors of uncertainty, and exacerbated them with the instability of the macroeconomic environment (rising inflation, rising interest rates on loans, and high residential indebtedness).

We have to take into account the time factor in relation to the willingness to have a child; therefore, we also asked respondents about their short, medium, and long-term intentions. When asked how many children are planned for the next one to two years (i.e., in the short term), we obtained a mean value of 1.58 (with a standard deviation of 0.74). Over a three to five-year period (medium term), respondents would like an average of 1.73 children (with a standard deviation of 0.67). Looking ahead to a period longer than five years, respondents plan an average of 1.97 children (with a standard deviation of 0.61). The values obtained are slightly skewed due to the fact that the possible answers are “I do not plan a child” (0), “I want one child” (1), “I want two children” (2) and “I want three or more children” (3); that is, a value of three indicated an intention to have three or possibly more children. According to the latest data, the fertility rate in Hungary in 2021 was 1.59, according to the Hungarian Central Statistical office-KSH) [2], slightly below the European average (1.61) (Statista) [3].

Dommermuth et al. [60], using data from Norway based on the Theory of Planned Behavior, found empirically that the reported duration of childbearing is relevant for actual childbearing, but that the patterns of childbearing behaviour are slightly different for respondents who were childless at the time of the interview compared to those who already had children. On the whole, those who were childless were less likely to have realised their intention to have children than those who were already parents; presumably because the former underestimated the difficulties associated with realising their intentions. In our study, we interviewed university students, most of whom were still childless; therefore, the result that their intention to have children is higher than the fertility rate measured by recent data is justified by the theory of planned behaviour.

## Conclusions

Our empirical research suggests that among university students of potential childbearing age, the impact of government financial instruments (tax credits, and housing subsidies) on their childbearing decisions is indirect and weak. Pro-birth policy elements, including tax incentives and subsidies through the housing subsidy scheme, have a moderate effect on the living conditions of families with children (as subsidies through the housing subsidy scheme are mostly absorbed by the housing market price increase), but have only a weak effect on the willingness of childbearing.

At the same time, young people of childbearing age (although they tend to think about having children after they have completed their tertiary education) do not fear that having children will reduce their leisure time or put a brake on their career. But the fact that they still have a low propensity to have children, and the responses we have received, suggest that they attach greater importance to the stability of their relationships and the socio-economic stability, democratic and environmental conditions of the country, i.e. the liveability of the country.

Our findings are consistent with the research of Moeeni et al. [61], who found that in their country with low fertility rates, women’s propensity to have children is mostly determined by the norms of their social community. Our research suggests that the government should aim to create a stable, inclusive environment conducive to childbearing by maintaining family policy incentives in the long term (not only by supporting childbearing but also by improving the infrastructural aspects of childrearing), by promoting family-friendly jobs and by investing in human capital.

A further direction of research on this topic we propose is a deeper exploration of the causal links between the elements of socio-cultural inclusiveness of childbearing, according to the income/regional stratification of the population under study; and the formulation of corresponding economic policy recommendations.

## Supporting information

S1 Data (CSV)
